# Coexistence of Small-Cell Lung Cancer and Gastrointestinal Malignancies: A Narrative Systematic Review of Case Reports

**DOI:** 10.7759/cureus.92393

**Published:** 2025-09-15

**Authors:** Ryuichi Ohta, Natsumi Yamamoto, Kaoru Tanaka, Chiaki Sano, Hidetoshi Hayashi

**Affiliations:** 1 Department of Community Care, Unnan City Hospital, Unnan, JPN; 2 Department of Medical Oncology, Kindai University Faculty of Medicine, Osaka, JPN; 3 Department of Community Medicine Management, Faculty of Medicine, Shimane University, Izumo, JPN; 4 Department of Medical Oncology, Kindai University Faculty of Medicine, Sayama, JPN

**Keywords:** case reports, gastrointestinal neoplasms, metachronous neoplasms, multiple primary neoplasms, small cell lung carcinoma, synchronous neoplasms

## Abstract

The coexistence of small-cell lung cancer (SCLC) and primary gastrointestinal (GI) malignancies is an exceptionally rare phenomenon that complicates diagnosis and treatment. We conducted a systematic review of published case reports to better understand the clinical characteristics, therapeutic approaches, and outcomes of these patients. A comprehensive search of major databases up to April 2025 identified six eligible reports describing patients diagnosed with both SCLC and distinct GI cancers, including gastric, duodenal, rectal, and jejunal tumors, as well as one patient with multiple primary malignancies involving rectosigmoid adenocarcinoma, renal cell carcinoma, and prostate adenocarcinoma in addition to SCLC. The patients were predominantly older males with heavy smoking histories, and most cases were extensive-stage SCLC at presentation. Treatment varied widely and included platinum-based chemotherapy, immune checkpoint inhibitors, EGFR-targeted therapy, surgery, and supportive care, with clinical outcomes ranging from a few months to more than one year of survival. Several patients showed partial or complete responses to systemic therapy, and in one case, the GI malignancy regressed following chemotherapy given for SCLC, suggesting overlapping chemosensitivity. However, other patients experienced delayed diagnosis of the second malignancy or limited survival due to disease progression or comorbidities. This synthesis demonstrates that distinguishing dual primaries from metastatic disease is a critical challenge in clinical practice and highlights the need for thorough evaluation in patients with atypical or persistent symptoms. Although data remain limited, awareness of this rare coexistence may help clinicians avoid misclassification and tailor multidisciplinary treatment strategies to improve outcomes.

## Introduction and background

Small-cell lung cancer (SCLC) accounts for approximately 13%-15% of all lung cancers and is characterized by rapid proliferation, early dissemination, and poor prognosis despite initial responsiveness to chemotherapy and radiotherapy [1]. It arises from neuroendocrine cells and is most frequently associated with heavy smoking [2]. While distant metastases to organs such as the brain, liver, bone, and adrenal glands are common, the coexistence of SCLC with other primary malignancies, particularly gastrointestinal (GI) cancers, is rare [2,3].

Multiple primary malignancies (MPMs) have become more frequently diagnosed due to advances in diagnostic imaging, cancer surveillance, and prolonged survival of cancer patients. MPMs are defined as the presence of more than one histologically distinct primary tumor in the same individual, either synchronously (within six months of each other) or metachronously (diagnosed more than six months apart) [4,5]. Among these, the coexistence of primary GI cancers such as gastric, colorectal, or duodenal cancer with SCLC remains exceptionally uncommon, often limited to case reports and small institutional case series [6].

The clinical presentation of dual malignancies poses significant diagnostic and therapeutic challenges [7]. Differentiating between metastasis and a second primary tumor is often difficult, particularly in smokers with advanced-stage SCLC who present with GI symptoms or incidental findings [6]. Moreover, treatment prioritization becomes complex due to differing tumor biology, prognosis, and therapy responsiveness [8]. While SCLC typically mandates systemic chemotherapy early on, GI tumors may benefit from surgical resection or targeted therapies depending on histology and staging [1].

To date, no systematic review has synthesized the clinical features, diagnostic considerations, treatment decisions, and outcomes of patients diagnosed with both SCLC and primary GI malignancies [9,10]. Understanding such rare clinical scenarios may improve clinician awareness, prevent diagnostic delays, and guide multidisciplinary decision-making. Therefore, this systematic review aims to evaluate the reported cases of synchronous or metachronous SCLC and GI malignancies, focusing on patient characteristics, tumor histology, diagnostic sequence, therapeutic strategies, and clinical outcomes. By aggregating the available evidence, we seek to provide a comprehensive reference that informs clinical practice and highlights areas requiring further investigation.

## Review

Methodology

Protocol and Registration

This systematic review was conducted in accordance with the Preferred Reporting Items for Systematic Reviews and Meta-Analyses (PRISMA) 2020 guidelines [11]. The protocol was prospectively registered with the International Prospective Register of Systematic Reviews (PROSPERO) under the registration number (CRD420251074092). The registered protocol outlines the objectives, eligibility criteria, data extraction process, risk of bias assessment tools, and planned synthesis strategy for evaluating the clinical features and management of patients with coexisting SCLC and GI malignancies.

Eligibility Criteria and Search Strategy

Studies were selected based on the following inclusion criteria: case reports of patients diagnosed with pathologically confirmed SCLC; coexistence of a histologically confirmed primary GI malignancy, including cancers of the stomach, small intestine, colon, rectum, or duodenum; and reports published in English with accessible full text and sufficient clinical detail, including treatment and outcomes. We excluded cases involving metastases from SCLC to the gastrointestinal tract or vice versa without confirmation of two distinct primaries, as well as non-English articles, conference abstracts without full text, and review articles. A systematic search was conducted in PubMed, Embase, and Web of Science for articles published from January 1980 to April 2025. The following search terms and Boolean operators were used: ("small cell lung cancer" OR "SCLC") AND ("gastrointestinal cancer" OR "gastric cancer" OR "colorectal cancer" OR "duodenal cancer" OR "intestinal cancer") AND ("synchronous" OR "metachronous") AND ("case report"). Reference lists of all included articles were hand-searched to identify additional eligible studies.

Data Extraction and Management

A standardized data extraction sheet was developed and piloted. The following variables were extracted from each included case report: demographic data (age, sex, smoking history); cancer-related data (site and histological type of GI malignancy, site and stage of SCLC, timing as synchronous or metachronous, and diagnostic methods); treatment details (chemotherapy, radiotherapy, surgery, targeted therapy, and immunotherapy); and outcomes (treatment response, survival time, recurrence, and cause of death). Two independent reviewers (RO and NY) conducted the screening, full-text review, and data extraction. Discrepancies were resolved through discussion or third-party adjudication.

Risk of Bias Assessment

Given the inclusion of case reports, we used the Joanna Briggs Institute (JBI) Critical Appraisal Checklist for case reports to assess methodological quality [12]. Each report was evaluated across eight domains, including patient history clarity, diagnostic criteria, intervention description, follow-up, and conclusions. Studies fulfilling ≥6 criteria were considered methodologically sound.

Outcomes and Data Synthesis

Due to the small number of cases and the inherent heterogeneity in cancer types, clinical context, and treatments, a meta-analysis was not feasible. Instead, we conducted a qualitative narrative synthesis. The primary outcomes of interest were the type and histology of GI malignancy associated with SCLC, the timing of dual diagnosis (synchronous vs. metachronous), the therapeutic approaches used (priority, sequencing, and modality), and the clinical outcomes, including survival, response to treatment, and complications. Findings were summarized in tabular and textual formats to highlight patterns, common challenges, and implications for practice.

Results

Study Selection: PRISMA Flow Diagram

**Figure 1:**
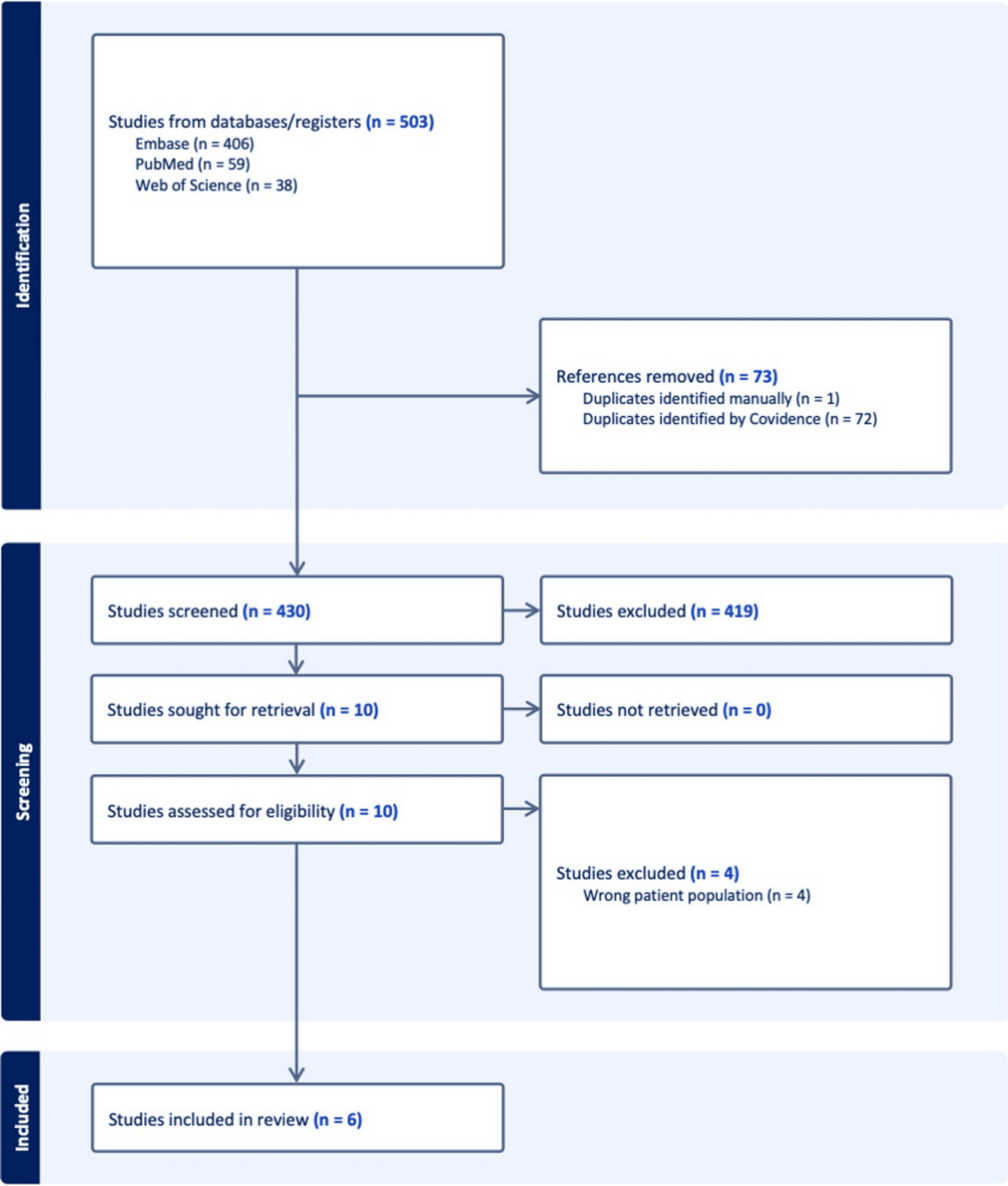
PRISMA 2020 flow diagram. PRISMA, Preferred Reporting Items for Systematic Reviews and Meta-Analyses

A total of 503 records were identified through electronic database searches: Embase (*n* = 406), PubMed (*n* = 59), and Web of Science (*n* = 38). After removing 73 duplicates - 72 identified through Covidence and one manually - 430 unique records remained for screening. Of these, 419 were excluded based on titles and abstracts due to irrelevance, including non-case reports, metastatic gastrointestinal involvement without dual primaries, or unrelated cancer types. The remaining 11 full-text articles were sought for retrieval and successfully obtained. Upon full-text review, four studies were excluded for involving the wrong patient population (e.g., lacking pathological confirmation of dual primaries). Finally, six case reports met all predefined inclusion criteria and were included in the systematic review. The complete PRISMA 2020 flow diagram detailing the selection process is presented in Figure 1.

Study Characteristics

This systematic review included six single-patient case reports published between 1986 and 2025, all describing patients diagnosed with SCLC in conjunction with a histologically distinct primary GI malignancy. These reports were drawn from various countries, including Japan (*n* = 3), China (*n* = 1), the United States (*n* = 1), and the Czech Republic (*n* = 1), reflecting the global recognition of this rare oncologic coexistence. In all cases, the SCLC diagnosis was pathologically confirmed, with one case involving combined small-cell lung cancer (CSCLC), which included an enteric-type adenocarcinoma component within the same lung tumor. This histologic combination highlights the morphologic heterogeneity that may be seen in lung cancers with mixed neuroendocrine and glandular differentiation (Table 1).

**Table 1:** Characteristics of the included articles. Timing (Sync/Meta): Synchronous: Both malignancies are diagnosed concurrently or within six months of each other; Metachronous: The second primary malignancy is diagnosed more than six months after the first. *Enteric adenocarcinoma (lung)* refers to a rare subtype of lung adenocarcinoma that histologically resembles gastrointestinal adenocarcinoma and was observed as a component of CSCLC in one case. The *quadruplicity* case includes four separate primary malignancies occurring over time: rectosigmoid adenocarcinoma, renal cell carcinoma, prostate adenocarcinoma, and SCLC. SCLC, small-cell lung cancer; CSCLC, combined small-cell lung cancer, defined as SCLC with additional histologic components (e.g., adenocarcinoma or squamous cell carcinoma); ED, extensive disease; LD, limited disease; GIST, gastrointestinal stromal tumor

Author (Year)	Country	Age/Sex	SCLC type	GI malignancy	Timing (Sync/Meta)
Sano et al. (1986) [13]	Japan	53/Male	ED-SCLC (oat cell carcinoma)	Signet ring cell gastric cancer	Synchronous
Kurishima et al. (2009) [14]	Japan	Not specified/Male	ED SCLC	Undifferentiated gastric cancer	Metachronous
Adwan et al. (2018) [15]	Czech Republic	66/Male	ED-SCLC (liver and mediastinal metastasis)	Rectosigmoid adenocarcinoma (quadruplicity)	Metachronous
Liu et al. (2020) [16]	USA	67/Male	LD-SCLC	Rectal adenocarcinoma and jejunal GIST	Synchronous
Wang et al. (2022) [17]	China	70/Female	LD-SCLC (combined SCLC and enteric adenocarcinoma)	Enteric adenocarcinoma (lung)	Synchronous
Ohta et al. (2025) [18]	Japan	80/Female	ED-SCLC	Primary duodenal adenocarcinoma	Synchronous

Risk of Bias Within Studies

This systematic review synthesized six published case reports that described patients diagnosed with both SCLC and histologically confirmed primary GI malignancies. Despite the heterogeneity of the cases, several consistent clinical patterns, diagnostic challenges, and therapeutic themes emerged.

Patient Characteristics and Timing of Diagnosis

The included patients ranged in age from 53 to 80 years, with a predominance of male patients (5 out of 6). Most had a significant smoking history, often exceeding 30 pack-years, reflecting the well-established link between tobacco exposure and the development of SCLC as well as certain GI cancers. Most cases (*n* = 4) were classified as having synchronous malignancies, meaning both cancers were diagnosed within a short time frame, typically during the initial staging or diagnostic work-up for one of the tumors. In two cases, the GI cancer was identified metachronously, either several months before or after the diagnosis of SCLC. In many instances, the initial clinical presentation was nonspecific, such as weight loss, abdominal discomfort, or anemia, and the second primary tumor was detected only after further investigation prompted by persistent or unexplained symptoms.

Tumor Sites and Histological Findings

The types of GI cancers identified across the six cases were diverse. Gastric adenocarcinoma was reported in two patients: one with signet ring cell histology and another with poorly differentiated adenocarcinoma. Duodenal adenocarcinoma was identified in a patient initially suspected to have GI metastasis from SCLC, later confirmed as a second primary. Colorectal involvement was described in two cases: one patient had synchronous rectal adenocarcinoma and a jejunal GIST; another had rectosigmoid adenocarcinoma as part of a rare tumor quadruplicity, which also included renal cell carcinoma and prostate adenocarcinoma. CSCLC with enteric-type adenocarcinoma was diagnosed in a patient whose lung mass exhibited dual histology. All cases demonstrated clear histopathological differentiation between the SCLC and the GI cancer, satisfying the Warren and Gates criteria for multiple primary malignancies.

Diagnostic Process and Challenges

In synchronous cases, the diagnosis of one tumor often prompted a thorough staging workup that incidentally revealed the second malignancy. This was commonly achieved through CT, PET-CT, upper/lower GI endoscopy, and biopsy. In metachronous cases, the second cancer was diagnosed later due to either new-onset symptoms or re-evaluation of persistent abnormalities.

Diagnostic confusion between metastatic spread versus synchronous primary was a recurrent challenge, particularly in cases with atypical imaging findings. One patient, for example, had a duodenal lesion initially interpreted as metastasis from SCLC, but subsequent endoscopic biopsy confirmed a primary duodenal adenocarcinoma. Another had a lung lesion with dual histology (SCLC and enteric adenocarcinoma), raising the question of mixed histogenesis or composite tumors.

Treatment Approaches and Strategies

The treatment plans reflected the biological aggressiveness of SCLC and the complexity of managing two distinct primary tumors. In five of six cases, the SCLC was treated with platinum-based chemotherapy, primarily carboplatin and etoposide, with or without radiotherapy. One patient also received durvalumab, an immune checkpoint inhibitor, which aligns with the contemporary standard of care for extensive-stage SCLC.

Management of the GI cancer varied. Surgical resection was performed in three cases: one for rectal adenocarcinoma and jejunal GIST, another for rectosigmoid adenocarcinoma, and a third for gastric cancer following response to systemic therapy. In contrast, in two elderly patients with poor performance status or advanced comorbidities, surgical treatment was not pursued due to concerns about perioperative risks. One notable case involved using gefitinib, an EGFR-tyrosine kinase inhibitor, in a patient with an EGFR p.L861Q mutation detected in the enteric component of a lesion. This targeted therapy was combined with platinum-doublet chemotherapy, resulting in a durable response.

Treatment sequencing was typically prioritized based on clinical urgency. In most cases, the SCLC was treated first due to its rapid progression and symptom burden, while GI malignancies were managed after stabilization or concurrently if clinically feasible.

Clinical Outcomes

Clinical outcomes varied depending on age, tumor burden, and treatment strategies. Partial or complete responses were observed in four patients, particularly in the SCLC component. One patient experienced complete histological remission of gastric cancer after chemotherapy intended for SCLC, suggesting potential cross-sensitivity of GI tumors to platinum-based regimens. In contrast, another patient developed ongoing GI bleeding due to an undiagnosed duodenal adenocarcinoma, underscoring the importance of timely recognition and tailored therapy for both malignancies.

Survival outcomes ranged from four months to over one year. One patient with quadruplicate tumors died due to progressive renal failure, likely exacerbated by cumulative treatment-related toxicity and advanced age. Another patient remained progression-free for 11 months on gefitinib and chemotherapy, highlighting the evolving role of precision oncology even in complex, multi-tumor contexts (Table 2).

**Table 2:** Treatment approaches and clinical outcomes. *Surgical resection* refers to curative-intent procedures such as low anterior resection or segmental bowel resection. *Partial response *and *stable disease* were reported by the original case authors based on imaging or histologic reassessment. *Supportive care *denotes palliative or non-curative management, often due to poor performance status or comorbidities. In one case, the GI tumor was histologically part of the pulmonary lesion (enteric adenocarcinoma within CSCLC) and thus not treated separately. SCLC, small-cell lung cancer; EP, etoposide plus a platinum agent (commonly cisplatin or carboplatin); CAV, cyclophosphamide, doxorubicin (Adriamycin), and vincristine - a second-line chemotherapy regimen for SCLC; Gefitinib, an epidermal growth factor receptor-tyrosine kinase inhibitor (EGFR-TKI) used in EGFR-mutated cancers; Durvalumab, a PD-L1 immune checkpoint inhibitor approved for use in extensive-stage SCLC after initial chemotherapy; GI cancer, Gastrointestinal malignancy, including gastric, colorectal, duodenal, and small intestinal tumors

Author (Year)	Treatment for SCLC	Treatment for GI cancer	Outcome
Sano et al. (1986) [13]	Carboplatin → CAV regimen	Gastric lesion resolved with chemotherapy (no surgery)	Partial response: no recurrence reported
Kurishima et al. (2009) [14]	Supportive care only	Gastric cancer treated initially; no further intervention	SCLC diagnosed years later; survival limited
Adwan et al. (2018) [15]	Chemotherapy + palliative radiotherapy	Supportive care; surgery for earlier cancers	Death from renal failure after multiple malignancies
Liu et al. (2020) [16]	EP (etoposide + cisplatin)	Surgical resection of rectal cancer and jejunal GIST	No recurrence at six-month follow-up
Wang et al. (2022) [17]	Gefitinib + EP (etoposide + etopside)	No separate GI treatment; part of the lung tumor	Stable disease at 11 months
Ohta et al. (2025) [18]	EP + durvalumab	Not resected; GI bleeding prompted diagnosis	Partial remission; died of progression at 7 months

Discussion

Summary of Main Findings

This systematic review summarizes six published case reports describing patients diagnosed with SCLC and a histologically distinct primary GI malignancy. The cases, published between 1986 and 2025, illustrate a rare but clinically significant intersection of two high-burden cancer types. The patients were predominantly older males with smoking histories, and most had extensive-stage SCLC at diagnosis. GI malignancies included gastric, duodenal, and rectosigmoid adenocarcinomas, as well as jejunal GIST and combined histology involving enteric adenocarcinoma within a lung tumor.

Synchronous presentation of both cancers occurred in four out of six cases, and the remaining two were diagnosed subsequently. Diagnostic processes frequently involved PET-CT and endoscopy, with some GI tumors initially misclassified as metastases. Treatment approaches prioritized SCLC in most cases, often using platinum-based chemotherapy. Surgical management of GI malignancies was applied selectively based on patient status. Clinical outcomes varied, with survival ranging from several months to over one year.

Comparison with Previous Literature

SCLC typically metastasizes to the liver, brain, and bone, but its coexistence with a second primary GI malignancy is rare and underreported. While MPMs occur in approximately 2%-5% of cancer patients, synchronous cases involving SCLC and GI cancers remain exceptional and are mostly documented through isolated case reports [6,19]. Previous studies have primarily focused on metachronous malignancies, often arising years after treatment of the index cancer [20,21]. In a Japanese series, most lung cancer patients with GI tumors were diagnosed metachronously, reflecting long-term surveillance and the impact of shared carcinogens such as smoking and alcohol [14]. In contrast, this review identified a predominance of synchronous cases, likely due to advances in imaging techniques, such as PET-CT, which enable the incidental detection of asymptomatic second primaries during initial staging.

Of particular note is the case of combined SCLC with enteric adenocarcinoma, a rare histological subtype resembling colorectal cancer. This underscores the complexity of distinguishing between metastasis, combined histology, and actual double primaries [17,22]. Similarly, the reported case of tumor quadruplicity (involving colorectal, renal, prostate, and SCLC) is exceedingly rare and highlights the potential role of environmental or occupational exposures.

Unlike most prior reports, several patients in this review responded to SCLC-directed platinum-based chemotherapy with regression in both lung and GI tumors, suggesting possible cross-sensitivity. Furthermore, the recent use of EGFR-TKIs and immune checkpoint inhibitors reflects a shift toward precision medicine; however, their role in managing synchronous malignancies remains to be defined [23]. In summary, this review complements existing literature by emphasizing the need for comprehensive evaluation at diagnosis. It raises new questions regarding biological overlap, diagnosis, and treatment strategies in patients with dual primary malignancies.

Clinical Implications

Clinicians managing patients with suspected or confirmed SCLC should remain vigilant for the possibility of second primary malignancies, particularly in those with persistent or unexplained GI symptoms. While it may be tempting to attribute all abnormal findings to SCLC metastasis, delayed or missed diagnoses of synchronous GI cancers can result in preventable morbidity [24]. Treatment planning should be multidisciplinary and individualized, balancing the aggressiveness of SCLC with the curability and resectability of the GI malignancy [25]. Cases such as the one involving EGFR-mutant enteric adenocarcinoma within CSCLC underscore the emerging role of molecular profiling in guiding therapy. Moreover, partial or complete responses of GI tumors to SCLC-directed chemotherapy in several cases raise essential questions about overlapping chemosensitivity and treatment prioritization.

Limitations

Several limitations should be acknowledged. First, the review is based solely on case reports, which inherently carry risks of publication bias, incomplete reporting, and lack of long-term follow-up. Second, the small sample size limits the generalizability of findings. Third, variations in diagnostic techniques, staging criteria, and therapeutic options across decades may have influenced management and outcomes. Finally, due to the narrative nature of available data, meta-analytic synthesis was not feasible.

Future Directions

Further research is needed to better understand the incidence, risk factors, and optimal management strategies for SCLC patients with synchronous or metachronous GI malignancies. The development of national or international registries could facilitate systematic data collection on rare multiple primary cancer scenarios. Studies examining the molecular and genetic basis of dual tumor development may also yield insights into common etiologic pathways, particularly in smokers. Additionally, guidelines for working up suspected second primaries in newly diagnosed SCLC patients may help standardize care.

## Conclusions

The coexistence of SCLC and primary GI malignancies is rare and has been described only in isolated case reports. In this review of six cases, we observed variability in the timing of dual diagnoses, frequent diagnostic uncertainty in distinguishing synchronous or metachronous primaries from metastatic disease, and diverse therapeutic approaches shaped by patient status and tumor characteristics. Reported outcomes ranged from a few months to more than one year, underscoring the heterogeneity of these cases rather than establishing any consistent prognostic pattern. These findings offer descriptive insights into the clinical challenges of managing patients with SCLC and highlight the importance of careful evaluation when GI symptoms arise. While no definitive conclusions can be drawn, these reports collectively suggest that recognizing the possibility of multiple primaries may help avoid misclassification and guide more tailored clinical decision-making.
